# 1*H*-Indole-3-carbaldehyde

**DOI:** 10.1107/S1600536812040573

**Published:** 2012-10-13

**Authors:** C. S. Dileep, M. M. M. Abdoh, M. P. Chakravarthy, K. N. Mohana, M. A. Sridhar

**Affiliations:** a Department of Studies in Physics, Manasagangotri, University of Mysore, Mysore 570 006, India; b Department of Physics, Faculty of Science, An Najah National University, Nabtus, West Bank, Palestinian Territories; c Department of Studies in Chemistry, Manasagangotri, University of Mysore, Mysore 570 006, India

## Abstract

In the title compound, C_9_H_7_NO, the benzene ring forms a dihedral angle of 3.98 (12)° with the pyrrole ring. In the crystal, N–H⋯O hydrogen bonds links the mol­ecules into chains which run parallel to [02-1].

## Related literature

For a related structure, see: Rizal *et al.* (2008 ▶).
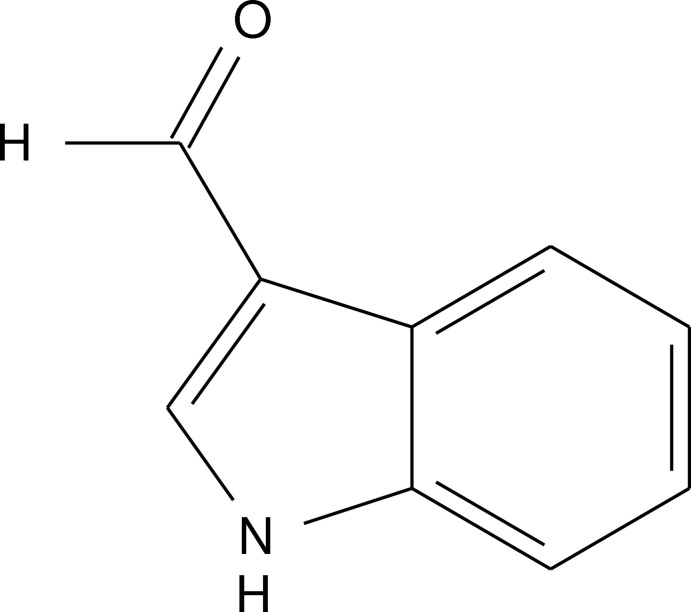


## Experimental

### 

#### Crystal data

C_9_H_7_NO
*M* *_r_* = 145.16
Orthorhombic, 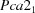

*a* = 14.0758 (9) Å
*b* = 5.8059 (4) Å
*c* = 8.6909 (5) Å
*V* = 710.24 (8) Å^3^
*Z* = 4
Mo *K*α radiation
μ = 0.09 mm^−1^
*T* = 293 K
0.30 × 0.20 × 0.20 mm

#### Data collection

Bruker Kappa APEXII CCD diffractometer
3791 measured reflections
775 independent reflections
699 reflections with *I* > 2σ(*I*)
*R*_int_ = 0.024

#### Refinement

*R*[*F* ^2^ > 2σ(*F* ^2^)] = 0.029
*wR*(*F* ^2^) = 0.069
*S* = 1.08
775 reflections
109 parameters
1 restraint
H atoms treated by a mixture of independent and constrained refinement
Δρ_max_ = 0.11 e Å^−3^
Δρ_min_ = −0.09 e Å^−3^

### 

Data collection: *APEX2* (Bruker, 2004 ▶); cell refinement: *APEX2* and *SAINT* (Bruker, 2004 ▶); data reduction: *SAINT* and *XPREP* (Bruker, 2004 ▶); program(s) used to solve structure: *SHELXS97* (Sheldrick, 2008 ▶); program(s) used to refine structure: *SHELXL97* (Sheldrick, 2008 ▶); molecular graphics: *PLATON* (Spek, 2009) ▶; software used to prepare material for publication: *PLATON* ▶.

## Supplementary Material

Crystal structure: contains datablock(s) I, global. DOI: 10.1107/S1600536812040573/go2070sup1.cif

Structure factors: contains datablock(s) I. DOI: 10.1107/S1600536812040573/go2070Isup2.hkl

Supplementary material file. DOI: 10.1107/S1600536812040573/go2070Isup3.cml

Additional supplementary materials:  crystallographic information; 3D view; checkCIF report

## Figures and Tables

**Table 1:** Hydrogen-bond geometry (Å, °)

*D*—H⋯*A*	*D*—H	H⋯*A*	*D*⋯*A*	*D*—H⋯*A*
N1—H1*A*⋯O1^i^	0.94 (3)	1.92 (3)	2.831 (2)	165 (3)

Symmetry code: (i) 
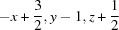
.

**Fig. 1.**
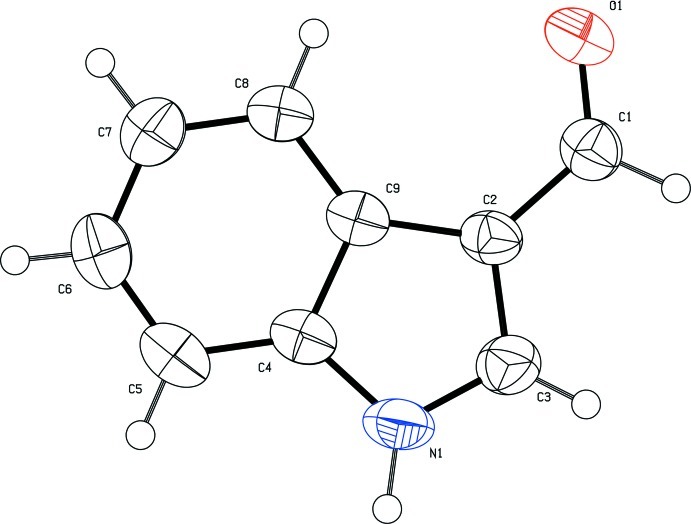
ORTEP of the title compound with the ellipsoids for non-H atoms are drawn at the 50% probability.

**Fig. 2.**
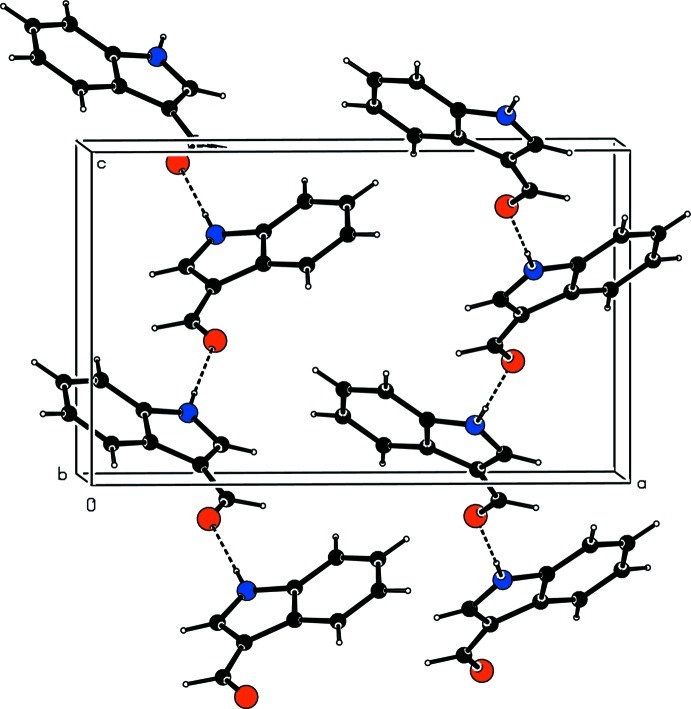
Molecular packing view of down the b axis. Dashed lines indicate hydrogen bonds.

## supplementary crystallographic information

### Comment

The molecule is shown with its labelling in Figure 1. The molecules are connected into one-dimensional chains by the N1–H1A···O1(3/2-*x*,-1+*y*,1/2+*z* hydrogen bond which links the molecules into one dimensional chains which run parellel to [2-10], Table 1 and Figure2.

### Experimental

Indole was converted to 1*H*-indole-3-carbaldehyde in the presence of DMF, POCl3, NaOH. 1*H*-indole-3-carbaldehyde was taken and recrystallized in methanol solvent. The purity of the compound is confirmed by the TLC. A little quantity of compound was taken again for recrystallization to get a pure crystal in methanol solvent medium.

### Refinement

H atoms were treated as riding atoms with C—H(aromatic), 0.93 Å with *U*_iso_ = 1.2Ueq(C). The H atoms attached to C1 and N1 were located on a difference map and refined isotropically. Friedel pairs were merged.

### Crystal data

**Table:**

C_9_H_7_NO	*Z* = 4
*M**_r_* = 145.16	*F*(000) = 304
Orthorhombic, *P**c**a*2_1_	*D*_x_ = 1.357 Mg m^−^^3^
Hall symbol: P 2c -2ac	Mo *K*α radiation, λ = 0.71073 Å
*a* = 14.0758 (9) Å	µ = 0.09 mm^−^^1^
*b* = 5.8059 (4) Å	*T* = 293 K
*c* = 8.6909 (5) Å	Block, colourless
*V* = 710.24 (8) Å^3^	0.30 × 0.20 × 0.20 mm

### Data collection

**Table:**

Bruker Kappa APEXII CCD diffractometer	699 reflections with *I* > 2σ(*I*)
Radiation source: fine-focus sealed tube	*R*_int_ = 0.024
Graphite monochromator	θ_max_ = 26.5°, θ_min_ = 2.9°
ω and φ scan	*h* = −17→16
3791 measured reflections	*k* = −6→7
775 independent reflections	*l* = −10→8

### Refinement

**Table:**

Refinement on *F*^2^	Secondary atom site location: difference Fourier map
Least-squares matrix: full	Hydrogen site location: inferred from neighbouring sites
*R*[*F*^2^ > 2σ(*F*^2^)] = 0.029	H atoms treated by a mixture of independent and constrained refinement
*wR*(*F*^2^) = 0.069	*w* = 1/[σ^2^(*F*_o_^2^) + (0.0297*P*)^2^ + 0.0895*P*] where *P* = (*F*_o_^2^ + 2*F*_c_^2^)/3
*S* = 1.08	(Δ/σ)_max_ = 0.002
775 reflections	Δρ_max_ = 0.11 e Å^−^^3^
109 parameters	Δρ_min_ = −0.09 e Å^−^^3^
1 restraint	Extinction correction: *SHELXL*, Fc^*^=kFc[1+0.001xFc^2^λ^3^/sin(2θ)]^-1/4^
Primary atom site location: structure-invariant direct methods	Extinction coefficient: 0.031 (5)

### Special details

**Table:**

Geometry. All e.s.d.'s (except the e.s.d. in the dihedral angle between two l.s. planes) are estimated using the full covariance matrix. The cell e.s.d.'s are taken into account individually in the estimation of e.s.d.'s in distances, angles and torsion angles; correlations between e.s.d.'s in cell parameters are only used when they are defined by crystal symmetry. An approximate (isotropic) treatment of cell e.s.d.'s is used for estimating e.s.d.'s involving l.s. planes.
Refinement. Refinement of *F*^2^ against ALL reflections. The weighted *R*-factor *wR* and goodness of fit *S* are based on *F*^2^, conventional *R*-factors *R* are based on *F*, with *F* set to zero for negative *F*^2^. The threshold expression of *F*^2^ > σ(*F*^2^) is used only for calculating *R*-factors(gt) *etc*. and is not relevant to the choice of reflections for refinement. *R*-factors based on *F*^2^ are statistically about twice as large as those based on *F*, and *R*- factors based on ALL data will be even larger.

### Fractional atomic coordinates and isotropic or equivalent isotropic displacement parameters (Å^2^)

**Table:**

	*x*	*y*	*z*	*U*_iso_*/*U*_eq_	
C1	0.76788 (16)	0.4092 (3)	−0.0642 (3)	0.0443 (5)	
C2	0.72368 (14)	0.2453 (4)	0.0341 (3)	0.0401 (5)	
C3	0.76781 (17)	0.0501 (4)	0.0871 (3)	0.0489 (6)	
H3	0.8297	0.0074	0.0628	0.059*	
C4	0.62446 (14)	0.0451 (3)	0.1907 (3)	0.0418 (5)	
C5	0.54556 (17)	−0.0080 (4)	0.2797 (3)	0.0500 (6)	
H5	0.5427	−0.1430	0.3371	0.060*	
C6	0.47250 (16)	0.1462 (4)	0.2795 (3)	0.0535 (6)	
H6	0.4189	0.1166	0.3388	0.064*	
C7	0.47646 (15)	0.3467 (4)	0.1924 (3)	0.0526 (6)	
H7	0.4255	0.4487	0.1951	0.063*	
C8	0.55405 (16)	0.3976 (4)	0.1024 (3)	0.0449 (5)	
H8	0.5555	0.5310	0.0433	0.054*	
C9	0.63026 (14)	0.2450 (3)	0.1019 (2)	0.0370 (5)	
N1	0.70988 (14)	−0.0700 (3)	0.1783 (2)	0.0510 (5)	
O1	0.72964 (12)	0.5795 (2)	−0.1176 (2)	0.0565 (5)	
H1	0.8360 (16)	0.376 (4)	−0.086 (3)	0.054 (6)*	
H1A	0.7253 (17)	−0.204 (4)	0.233 (4)	0.073 (8)*	

### Atomic displacement parameters (Å^2^)

**Table:**

	*U*^11^	*U*^22^	*U*^33^	*U*^12^	*U*^13^	*U*^23^
C1	0.0516 (13)	0.0432 (10)	0.0382 (12)	−0.0002 (9)	0.0027 (11)	−0.0014 (10)
C2	0.0514 (11)	0.0368 (9)	0.0323 (10)	0.0032 (9)	0.0004 (9)	0.0009 (8)
C3	0.0557 (14)	0.0476 (11)	0.0435 (14)	0.0109 (11)	0.0050 (11)	0.0022 (10)
C4	0.0557 (12)	0.0341 (9)	0.0354 (11)	−0.0017 (8)	−0.0038 (10)	0.0013 (10)
C5	0.0666 (14)	0.0435 (12)	0.0399 (13)	−0.0135 (11)	−0.0021 (12)	0.0050 (10)
C6	0.0499 (13)	0.0624 (14)	0.0484 (14)	−0.0136 (11)	0.0036 (11)	−0.0011 (12)
C7	0.0466 (12)	0.0576 (13)	0.0536 (15)	0.0036 (10)	−0.0017 (12)	−0.0041 (14)
C8	0.0512 (12)	0.0406 (10)	0.0428 (13)	0.0013 (9)	−0.0046 (11)	0.0037 (9)
C9	0.0473 (11)	0.0344 (10)	0.0293 (10)	−0.0027 (8)	−0.0052 (9)	−0.0017 (8)
N1	0.0680 (12)	0.0395 (9)	0.0455 (11)	0.0102 (8)	0.0016 (10)	0.0113 (10)
O1	0.0658 (11)	0.0442 (8)	0.0596 (11)	−0.0002 (7)	0.0058 (8)	0.0163 (8)

### Geometric parameters (Å, º)

**Table:**

C1—O1	1.218 (2)	C5—C6	1.363 (3)
C1—C2	1.422 (3)	C5—H5	0.9300
C1—H1	1.00 (2)	C6—C7	1.390 (3)
C2—C3	1.372 (3)	C6—H6	0.9300
C2—C9	1.441 (3)	C7—C8	1.375 (3)
C3—N1	1.334 (3)	C7—H7	0.9300
C3—H3	0.9300	C8—C9	1.391 (3)
C4—N1	1.380 (3)	C8—H8	0.9300
C4—C5	1.388 (3)	N1—H1A	0.94 (2)
C4—C9	1.396 (3)		
			
O1—C1—C2	125.4 (2)	C5—C6—C7	121.4 (2)
O1—C1—H1	120.6 (14)	C5—C6—H6	119.3
C2—C1—H1	114.0 (14)	C7—C6—H6	119.3
C3—C2—C1	123.8 (2)	C8—C7—C6	121.4 (2)
C3—C2—C9	105.93 (19)	C8—C7—H7	119.3
C1—C2—C9	130.22 (19)	C6—C7—H7	119.3
N1—C3—C2	110.8 (2)	C7—C8—C9	118.5 (2)
N1—C3—H3	124.6	C7—C8—H8	120.7
C2—C3—H3	124.6	C9—C8—H8	120.7
N1—C4—C5	129.30 (19)	C8—C9—C4	118.84 (19)
N1—C4—C9	107.95 (18)	C8—C9—C2	134.75 (19)
C5—C4—C9	122.65 (19)	C4—C9—C2	106.31 (18)
C6—C5—C4	117.2 (2)	C3—N1—C4	109.04 (17)
C6—C5—H5	121.4	C3—N1—H1A	126.4 (16)
C4—C5—H5	121.4	C4—N1—H1A	124.4 (16)
			
O1—C1—C2—C3	177.2 (2)	N1—C4—C9—C8	−176.6 (2)
O1—C1—C2—C9	−4.9 (4)	C5—C4—C9—C8	0.1 (3)
C1—C2—C3—N1	179.3 (2)	N1—C4—C9—C2	0.2 (2)
C9—C2—C3—N1	1.0 (3)	C5—C4—C9—C2	176.9 (2)
N1—C4—C5—C6	175.0 (2)	C3—C2—C9—C8	175.3 (2)
C9—C4—C5—C6	−0.9 (3)	C1—C2—C9—C8	−2.8 (4)
C4—C5—C6—C7	0.7 (4)	C3—C2—C9—C4	−0.7 (2)
C5—C6—C7—C8	0.3 (4)	C1—C2—C9—C4	−178.9 (2)
C6—C7—C8—C9	−1.1 (3)	C2—C3—N1—C4	−0.8 (3)
C7—C8—C9—C4	0.9 (3)	C5—C4—N1—C3	−176.0 (2)
C7—C8—C9—C2	−174.8 (2)	C9—C4—N1—C3	0.4 (2)

### Hydrogen-bond geometry (Å, º)

**Table:**

*D*—H···*A*	*D*—H	H···*A*	*D*···*A*	*D*—H···*A*
N1—H1*A*···O1^i^	0.94 (3)	1.92 (3)	2.831 (2)	165 (3)

Symmetry code: (i) −*x*+3/2, *y*−1, *z*+1/2.
